# Statutory and policy-based eco-disaster risk reduction in SADC member states

**DOI:** 10.4102/jamba.v16i2.1799

**Published:** 2024-10-30

**Authors:** Alfredo A. Covele, Dewald van Niekerk, Dirk Cilliers

**Affiliations:** 1 African Centre for Disaster Studies, Faculty of Natural and Agricultural Sciences, North-West University, Potchefstroom, South Africa; 2 Department of Electrotech, Faculty of Engineer, Eduardo Mondlane University, Maputo, Mozambique; 3 Department of Geo- and Spatial Sciences, Faculty of Natural and Agricultural Science, North-West University, Potchefstroom, South Africa

**Keywords:** disasters risk, disaster risk reduction, policies, SADC, Eco-DRR

## Abstract

Effective legislative framework is the cornerstone of managing hazards and disasters because they have become policy problems of global and local concern. This research study aims at understanding the implementation, strengths and gaps of policies related to Eco-DRR in SADC member states. In particular, attempts to critically analyse the making of DRM policies, as well as the variables underpinning these policies, given the high level of disaster losses. A literature survey was conducted to contextualise and conceptualise statutory and policy-based Eco-DRR. Academic literature on Eco-DRR and related policies, journal articles and related policies, official documents in SADC states including policies, acts, legislations, strategies, frameworks and plans were consulted. The analysis revealed that the Eco-DRR approaches have not yet been mainstreamed as part of standards of DRM in most of SADC member states, opting largely on ad hoc practice. Short-term plans and/or strategies don’t help to articulate funding and programme priorities. In addition, irregular updating of policies in some member states and a lack of following up mechanisms were noted.

**Contribution:**

To change this reality, it is necessary to include Eco-DRR in strategies and/or plans and to standardise ecosystem-based measures for reducing disaster risks. Additionally, there is an urgent need for empowerment of the existing institutions and creation of networks that are driven by SADC institutions. Overall, it is evident that there is a regional interest and demand to apply and standardise ecosystem-based approaches and natural or green infrastructure solutions toward Eco-DRR.

## Introduction

Many recent studies within disaster risk reduction (DRR) (e.g., Forino et al. 2017; Nemakonde & Van Niekerk 2017; Pilli-Sihvola & Vaatainen-Chimpuku 2016; Whelchel et al. 2018) look intrinsically at connecting DRR with climate change adaptation (CCA). This connection is recorded by McVittie et al. (2018) as the required bridge to minimise the consequences of extreme events and increase resilience to disasters, especially among vulnerable people. Both DRR and CCA are bound by effective and sustainable management of the ecosystem (Murti & Mathez-Stiefel 2019; PEDRR 2010; Renaud et al. 2016). Ecosystem-based disaster risk reduction (Eco-DRR) entails the management of the ecosystem for DRR which encompasses sustainability, conservation and restoration as variables to reduce disaster risk with the objective of achieving sustainable and resilient development (Estrella & Saalismaa 2013; Murti & Mathez-Stiefel 2019). Management of ecosystem for DRR relies on protection and restoration of natural ecosystem to reduce the vulnerability of society to stresses associated with climate change (Fang et al. 2014; Pramova et al. 2012; Reid 2011; Sutherby & Tomaszewski 2018). Many scholars point to Eco-DRR as involving multiple sectors and transdisciplinarity, calling for convergence of stakeholders’ interests and consensus (Cheema, Mehmood & Imra 2016; Murti & Mathez-Stiefel 2019; Takeuchi et al. 2016).

Development of policies, legislations and institutional frameworks are key activities towards achieving effective Eco-DRR (Chipangura, Van Niekerk & Van Der Waldt 2017; Williams 2011). Manyena et al. (2013), in a similar vein, argue that an effective legislative framework is the cornerstone of managing hazards and disasters because they have become policy problems of global and local concern. The effectiveness of Eco-DRR is largely dependent on systematic formulation of policy strategies, legal provisions, institutions, and its roles and responsibilities in dealing with disasters (Nepal, Khanal & Pangali Sharma 2018). Specifically, laws and regulations are imperative tools that offer solid foundation for building communities’ resilience, as they are effectively needed to guarantee the reduction of existing risk posed by hazards, preventing new risks from even arising and making people feel safer (Ahmed 2013; Mashi, Oghenejabor & Inkani 2019).

This suggests that disasters are partly produced by weaknesses in how legislative frameworks define objectives and protocols as well as in allocating mandates and duties to different actors (Jones et al. 2014; Manyena et al. 2013; Williams 2011). Although many countries have established empowering statutory, institutional and legislative frameworks over the past 20 years, some countries lag behind and have high level of disaster losses (Chipangura et al. 2017; Jones et al. 2014; Van Niekerk 2015; Williams 2011).

This research article aims at providing an identification of policies, legislations, strategies, frameworks and plans related to Eco-DRR in Southern Africa Development Community (SADC) member states. Wider-ranging literature related to policies, strategies, framework and plans supporting Eco-DRR have been conducted in the scientific community. Cheema et al. (2016) provided a historical analysis of the disaster management structure, policies and institution in Pakistan. In addition, Zafarullah and Huque (2018), presented a study that aimed at assessing some of the existing policies and strategies in selected South Asian countries. Recently, Ashu and Van Niekerk (2019) conducted a similar study in Cameroon to critically analyse the existing policies and legislations governing disaster risk management (DRM). To the best of our knowledge, no study of this nature has ever been conducted for the whole of SADC region member states that specifically focussed on Eco-DRR at national level. There is also a general lack of critical analysis of the effectiveness of the previous Eco-DRR policies, processes and institutional structures (Cheema et al. 2016; Manyena et al. 2013). Therefore, this research paper will also address this gap by performing an in-depth analysis of the implementation, strengths and limitations of the existing policies as well as by identifying the variables underpinning the policies related to Eco-DRR in the SADC region.

## Background

The SADC is an economic and political organisation founded with the objective of providing its member states with sustainable development and stable economic growth, while promoting peace and alleviating hunger for an enhanced standard and quality of life, especially for the socially disadvantaged (SADC 2010a; Tau, Van Niekerk & Becker 2016). The SADC was established in 1992 and initially comprised of 15 member states, namely, Angola, Botswana, the Democratic Republic of Congo, Lesotho, Madagascar, Malawi, Mauritius, Mozambique, Namibia, Seychelles, South Africa, Eswatini, Tanzania, Zambia and Zimbabwe (Tau et al. 2016). During the 38th Ordinary SADC Summit in August 2018, the Union of Comoros deposited its Instrument of Accession and became the 16th full member of SADC (SADC 2018a).

**FIGURE 1:**
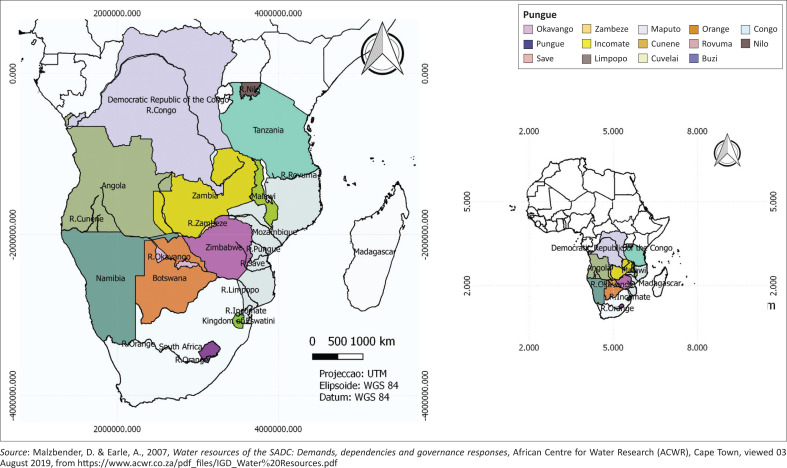
Southern Africa Development Community member states and major rivers.

In the southern African region, characteristics such as sharp orography, oceanic disparities coupled with high atmospheric dynamics are catalysts for the occurrence of extreme weather events and the annual variations in hydrological cycle (Fauchereau et al. 2003). Therefore, the region’s climate vulnerability hotspot is because of its erratic climatic regimes and high current and projected climate risk (Kapuka, Hlásny & Helmschrot 2022). The climate across the region varies substantially, with arid conditions in the west, Mediterranean conditions in the south-west and humid subtropical condition in the north and east. Tropical, extra-tropical and mid-latitude processes influence the region’s climate, with many remote processes driving annual and decadal climate variability (Daron et al. 2019). The most common extreme weather events are floods, large storms, droughts and wildfires (Davis-Reddy & Vincent 2017; Kruger 2016). These account for an estimated 67% of natural hazard-related deaths (Davis-Reddy & Vincent 2017). Another characteristic to consider is that the southern Africa region has 15 major river basins that are shared between two or more countries. In terms of size, they range from the Umbeluzi River Basin (5500 km^2^) that is shared by two countries, followed by the Zambezi River Basin (1 400 000 km^2^) which is shared by six SADC member states and finally the Congo River Basin (3 800 000 km^2^). The share of these watercourse systems has resulted in potential conflicts and creation of complex water rights (Malzbender & Earle 2007). Figure 1 shows the SADC member states and the major rivers.

According to Davis-Reddy and Vincent (2017), in the past four decades (1980–2015), SADC registered 491 climate disasters (meteorological, hydrological and climatological) that affected 140 million people with approximately 110 978 deaths and 2.47 million people displaced. The negative impact of the 2015/2016 El Niño event that induced drought, which was the worst in 35 years, affected 39 million people across the southern African region (SADC 2016).

The El Niño phenomenon is characteristic of the Pacific Ocean where patterns of warm water across the ocean’s surface cause changes in weather, thereby resulting in reduced rainfall and consequently drought. Its impacts are felt mostly in the eastern and southern African regions, and affect almost all sectors (water and sanitation, food security, agriculture, nutrition, energy, education, health and manufacturing). This causes most of the population to suffer and a contraction in the economy (FAO 2018).

In March 2019, the destructive Cyclone Idai significantly affected countries such as Mozambique, Madagascar, Malawi and Zimbabwe. It claimed the lives of about 700 people, destroyed the Mozambique’s port city of Beira, and flooded about 138 000 km^2^ of cropland (In on Africa [IOA] 2019). According to Davis-Reddy and Vincent (2017), the frequency of occurrence of these events (particularly floods, droughts, wildfires and storm surges) is likely to increase through the 21st century. In addition, the authors pointed out that this presents a significant challenge to the SADC DRM Unit (SADC DRRU) and the impact will expand to include infrastructure and transport, agriculture, health, tourism and insurance sectors, among others. This challenge is of extreme concern for the SADC DRRU as proven by the recent revised DRR Strategy for SADC & Plan of Action 2018–2030 as well as the SADC Regional Resilience Framework 2019 that states the regional efforts to improve partnerships and capacities for reducing risk and impacts of disasters as being of crucial importance. However, the lack of capacity and skills in many Member States and the over-reliance on the international assistance and funding system have caused slow progress in the implementation of regional, national and local plans of actions towards resilience building. There is a common understanding for a need of expeditious strengthening of the SADC DRRU to coordinate DRR programmes and facilitate the flow of early warning information in the sub-region (SADC 2010b, 2020).

## Theoretical underpinning: Policies related to ecosystem disaster risk reduction

The role of ecosystem-based DRR is globally recognised and tremendous developments have taken place. According to Uy and Shaw (2012), ecosystems provide a protective barrier against natural hazards. This is essential in mitigating the impact of natural hazards. Therefore, it is of paramount importance to keep them healthy, thus assisting vulnerable people’s resilience to natural hazards (Uy, Delfino & Shaw 2016). Moreover, they form the foundation for promoting CCA and DRR with the unified objective of attaining sustainable development while ensuring human well-being and security (PEDRR 2010). Recent years have been characterised with an increased interest in the research and implementation of policies related to ecosystem-based approaches for DRR (Renaud et al. 2016).

Despite the growing international support for Eco-DRR approaches, many countries lag behind in terms of mainstreaming them as part of the standard of disaster (risk) management policy and practice (Dhyani et al. 2018; Renaud et al. 2016; Whelchel et al. 2018). The mainstreaming of Eco-DRR approaches has been largely on an *ad hoc* basis and only a few vulnerable countries are acting because of the slow political processes in the international negotiations (Munang et al. 2013). This is because there is still large reliance on conventional engineering approaches, such as the use of retaining walls and groins, raised embankments to enhance protection and therefore manage disaster and climate risks (Whelchel et al. 2018). Whelchel et al. (2018) point out that the lack of standardised technical guidelines for the design and implementation of ecosystem-based measures such as maintenance, enhancement and restoration of ecosystem for reducing disaster risks has been a major obstacle for the engineering community to replicate and implement such measures. Another major reason is the failure to provide evidence on the cost effectiveness, equitable and sustainable means of securing climate adaptation and DRR benefits that result from investing in ecosystems (Emerton et al. 2016). Although the past decade has witnessed the emergence of guidelines for implementing Eco-DRR measures in way of how to restore and preserve ecosystem functions to DRR, rigorous testing and standardisation are required to ensure robustness. In addition, they are not easily accessible and are limited to certain types of ecosystems and hazards (Whelchel et al. 2018).

There are core elements in the implementation of the Eco-DRR which can be summarised in the following steps from the work of Partnership for Environment and Disaster Risk Reduction – PEDRR (2010):

- The first step consists of identifying the function provided by the ecosystems. This can either be natural hazard protection or mitigation;
- The second step merges Eco-DRR with sustainable development and livelihoods;
- The third step links the hard engineering approaches to the ecosystem-based options;
- The fourth step consists of performing a risk assessment on the climate change and extreme natural events and proposes measures to reduce their impacts on the above-selected ecosystem approaches;
- The fifth step is about being more integrating and functional. Here, the aim is to increase and/or enhance governance capability for Eco-DRR by involving multi-sector and multidisciplinary mechanisms or platforms. These platforms facilitate sharing of available data, and help to ensure scientific and technical rigour in designing and implementing ecosystem-based DRR initiatives;
- In the sixth step, the local stakeholders are informed and made part of the decision-making;
- The seventh and final step, makes use of the existing tools and instruments for the continual management and enhancement of the ecosystem and thus ensuring resilience and increased DRR value. These tools include the environmental assessment, integrated risk and vulnerability assessment, protected area management, integrated ecosystem management and community-based sustainable natural resources management.

Sustainable management, conservation and restoration of ecosystems form the pillars for achieving resilient and sustainable development (Dhyani et al. 2018; Schelchen et al. 2017; The World Bank 2010). Dhyani et al. (2018) consider these three variables (conservation, restoration and management) as contributing to framing effective policies in Eco-DRR.

Even after a successful implementation or consideration, Eco-DRR attains its true value when it is incorporated into policies and decisions that ensure systematic changes to minimise vulnerability to natural hazards (PEDRR 2010). In other words, it is necessary to link Eco-DRR to policy and institutional mandates to facilitate its implementation (Uy et al. 2016). Policies are intrinsically linked to objectives or course of action planned by the government on a particular subject (BES 2017). This implies that governments are the central actors in enabling appropriate Eco-DRR policies, and regulatory and implementation structures. This is true because being the government body responsible for the public safety, the governments ultimately have the mandate, capacity and resources to create, stimulate or undertake large-scale DRR initiatives. Moreover, it is at this level where political support is sought to integrate the initiatives into a national or local development plans (PEDRR 2010). Governments play a key role in ensuring planning and participation mechanisms, definition of policy, establishment of robust institutions, local authority capacity building and partnership between numerous stakeholders, including civil society, non-governmental organisations (NGOs) and the private sector (Diagne & Ndiaye 2014). Civil society also plays an important role in influencing large scale DRR implementation through society engagement and partnership (Birkland 2016; Handmer & Dovers 2007; Twigg 2015). Thus, Eco-DRR brings together several disciplines and sectors that ensure scientific and technical rigour in the design and implementation of initiatives. In short, it requires participatory management of DRR at transboundary, national, subnational and local levels.

Advancing policies and practices to incorporate new thinking on vulnerability, DRR and resilience are mentioned as major challenges faced by several countries around the world (Mashi et al. 2019; Twigg 2015). Allied to this is an assessment of existing Eco-DRR policies, legislations, strategies, frameworks and plans, which can help in framing and mainstreaming ecosystem-based strategies into effective policies, legislations, strategies, frameworks and plans.

## Research methods and design

The research aimed to answer three key questions: What are the policies, legislations, strategies, frameworks and plans related to Eco-DRR in the southern Africa member states; what are the gaps and strengths of implemented policies, strategies, frameworks, and plans related to Eco-DRR in the southern Africa member states; and what are the variables underpinning these policies and legislations? To answer these questions, a desktop analysis of SADC member states’ policies, legislations and framework was conducted to contextualise and conceptualise statutory and policy-based Eco-DRR in SADC member states. The consulted sources ranged from academic books on Eco-DRR and related policies, journal articles on Eco-DRR and related policies, official documents in SADC states such as policies, legislations, strategies, frameworks and plans. It also drew from various databases such as the PreventionWeb, ReliefWeb, SADC website, United Nations Office for DRR and Disaster Law. The combination of keywords such as disaster risk, ecosystem-based management, eco-disaster risk, Eco-DRR, policies in disaster risk and disaster profile in SADC were used for online searches. A decisive assessment of the authority of the literature was done mainly by cross-examining authors’ successions of arguments and conclusions.

## Results

Through the analysis and policy review for each SADC member states, the following summarisation was done:

- In the Republic of Angola, the main statutory instrument on disaster management is the Basic Civil Protection Law of 2003 (Law 28/03), which was amended by Law 14/20 of 22 May 2020. The main objective of the Basic Civil Protection Law is to reduce disaster risk through the development of relief action, prevention and training. The instrument also establishes a leading institutional cross-cutting body for DRR activities (IFRCR 2021).
- In addition, the national plan for preparation, resilience, response and recovery from natural hazards 2015–2017 was established through Presidential decree n° 29/16 of 01 of February 2016, but the new national plan is not published yet. Another Presidential decree n° 30/16 of 03 of February 2016 established the strategic plan for disaster prevention and risk reduction, with particularity of adopting risk transfer as DRM strategy. Some sectoral statutory instruments were established as Basic Environmental Law No 5/98, aiming to establish the mandatory licensing of activities that are likely to cause environmental impacts. The law of Forests, Fauna, Wild and Terrestrial Conservation Areas in 2007 aiming to ensure that the use of forests and wildlife terrestrial is guided by the relevant constitutional and international law principles, in particular the principles of sustainable development and environmental protection.
- In the Republic of Botswana, there is not a legislation dedicated to DRM (IFRCR 2021). The existing statutory instruments are the National Policy on Disaster Management (1996), the National Disaster Risk Management Plan (2009) and the National Disaster Risk Reduction Strategy 2013–2018. Environment, biodiversity and ecosystem aspects are in separate statutory instruments such as *Wildlife Conservation and National Parks Act (1998)*, Botswana Environment & Climate Change Law (2020).
- In Comoros, only one national response statutory instrument towards addressing Eco-DRR is available – the National Action Plan Against Desertification, 2013. The aim of the National Action Plan is to combat desertification, land degradation and drought.
- In the Democratic Republic of Congo (DRC), the National Strategy of Prevention and Reduction of Disaster Risk 2016–2023 was approved in 2017. The country’s National Adaptation Plan was finalised in 2006. The DRC’s CCA strategies focus on the preparation and strengthening of both institutions and institutional frameworks, improving responsible environmental management and adaptation efforts (The World Bank Group 2021). The Environment Protection Law was enacted in 2011 with the aim of promoting mainstreaming of environmental and sustainable development issues into all policies, plans and programmes across all relevant sectors.
- In Lesotho, DRM is regulated by the *Disaster Management Act No. 2 of 1997*, which was enacted to establish and regulate the Disaster Management Authority and other DRM institutions, as well as to make provision for the management of disaster including prevention, mitigation, preparedness, response and recovery (IFRCR 2021). Environment and ecosystem issues are regulated through separate documents such as Lesotho National Environment Policy of 1998 with aims to achieve sustainable livelihoods and development for Lesotho and ensure protection and conservation of the environment and sustainable development. In addition, the *Lesotho Environment Act No. 10 of 2008* was approved, which makes provision for the protection and management of the environment and conservation and sustainable utilisation of natural resources of Lesotho and for connected matters. In the same year, the Lesotho Forestry Policy 2008 was formulated with the aim to promote the use of trees in support of conservation and production of both arable agriculture and rangelands.
- In the Republic of Madagascar, the legal framework for DRR and management and the guide on DRR decision making is the National Disaster Risk Management Strategy 2016–2030. Other guiding statutory instruments on DRR include the National Risk and Disaster Management Strategy 2016–2020 and the National Risk and Disaster Management Policy 2015. Conservation, restoration and management of ecosystem and biodiversity are separately legislated in Forestry Legislation No 97-017; National Environment Action Plan 1991–2007 and the National Climate Change Policy (2015).
- The Republic of Malawi adopted DRM as one of the core focus areas of the Malawi Growth and Development Strategy (MGDS 2011–2016) and has taken various actions to support CCA. The Republic has developed the National Climate Change Management Policy, the National Climate Change Investment Plan (2013–2018) and the National Resilience Strategy (2012–2018) (Government of Malawi 2015). The main DRR statutory tool in Malawi is the National Disaster Risk Management Policy (2015), aiming to ensure that DRM is integrated in development planning by all sectors in the country. Other linked legislative frameworks and strategies to address Eco-DRR include the Malawi Constitution, *Environment Management Act of 1996*, the *Forestry Act of 1997*, the *Water Resources Act of 1969* and the *Town and Country Planning Act of 1988* (IUCN/PACO 2016).
- In the Republic of Mauritius, the legal framework addressing DRR is the *National Disaster Risk Reduction and Management Act 2016*. No other available legislative framework addresses the ecosystem and biodiversity adaptation, conservation and management.
- The Republic of Mozambique has implemented various DRM-related laws, policies, strategies and plans over the past 20 years (IDRL 2021). Mozambique had adopted the National Policy on Disaster Management early on in 1999, that was followed by the Master Plan for Prevention and Mitigation of Disaster 2006. The law establishing the legal framework for disaster management was adopted in 2014; that was replaced in 2020 by the Law on Disaster Risk Reduction and Management (Law 20/2020). In 2017, the National Disaster Risk Master Plan 2017–2030 was approved. Ecosystem Disaster Risk Reduction and biodiversity are regulated in the Conservation Law 2014 (Law 16/2014), aiming to establish the roles on protection, conservation and sustainable use of biological diversity; Environment Law 1997 (Law 20/1997); The *Forestry and Wildlife Act 1999 (Act 10/1999)*, covering the protection of the forest and the wildlife resources, as well as the sustainable forest resources, wildlife conservation, restoration and management.
- In the Republic of Namibia, the legal framework on DRM is the *Disaster Risk Management Act 10 of 2012*. In addition, the Namibian Government has published several implementing policies and regulations, including the Namibian National Disaster Risk Management Policy 2009; National Disaster Risk Management Plan 2011 and the Disaster Risk Management Regulation 2013 to provide for the implementation of the *DRM Act* (IDRL 2021). Protection, adaptation and management of ecosystem and biodiversity are regulated in Nature Conservation Ordinance Law of 1975 (Law 4/1975), *Environment Management Act of 2007* (Act 7/2007) and *Forestry Act of 2001* (Act 12/2001).
- In the Republic of Seychelles, the main legal framework addressing DRM is the *Disaster Risk Management Act 2014*, which establishes that a National Disaster Risk Management Plan and Strategy must be prepared. Seychelles also has other statutory instrument on biodiversity and ecosystem conservation, adaptation and management such as: The Coastal Management Plan 2019–2024, aiming to consolidate risk information and to provide a framework for its use for coastal management, adaptation and risk management; The National Climate Change Strategy 2009, aiming to mainstream climate change into sustainable development as national cross-sectoral programme addressing matters of policy, institutions, capacity building and civil society involvement (Urquhart & Lotz-Sisitka 2014); *The Environment Protection Act No. 9 of 1994*, aiming to prevent, control and abate environmental pollution; The *Conservation and Climate Adaptation Trust of Seychelles Act 18 of 2015*, which provides for establishment of the conservation and climate adaptation trust of Seychelles.
- In the Republic of South Africa, the *Disaster Management Act No. 57 of 2002* aims to provide for an integrated and coordinated disaster management policy that focusses on preventing and reducing disaster risk, mitigating the severity of disasters, disaster preparedness and response, as well as post-disaster recovery. Other statutory elements include the *Protected Areas Act of 2003* which allowed non-state lands to become protected areas; the *National Environmental Management Biodiversity Act No. 10 of 2004* which provides for the management and conservation of South Africa’s biodiversity within the framework of *National Environment Act No. 107 of 1998*, which provides for the protection of species and ecosystem that warranty national protection. In addition, there is a National Climate Change Adaptation Strategy of 2018, that provides a common vision of CCA and resilience for the country, and outlines priority areas for achieving these visions.
- In the Kingdom of Eswatini, the main guiding DRR instrument is the *Disaster Management Act No. 1, of 2006* with the aim to provide for the integration and coordination of disaster management in Eswatini. The country has also prepared the National Emergency Response, Mitigation and Adaptation Plan (NERMAP), which covers the period of January 2006 to March 2022 (IDRL 2021). Other instruments relevant to Eco-DRR range from National Climate Change Policy (2016), *Forest Preservation No. 14, of 1910* and *Natural Resources Act No. 72, of 1951*.
- In the United Republic of Tanzania, the DRR statutory framework is based on the *Disaster Management Act of 2015* with the aim of setting out a comprehensive legal framework for DRM. In addition, there is a Zanzibar Disaster Management Policy 2011 that provides situational analysis of the natural hazard risk and documents the linkages between disaster management and development policies. About Eco-DRR, there’s National Climate Change Strategy of 2012, aiming to enhance the technical, institutional and individual capacity of the country to address the impacts of climate change; *The Forest Act of 2002*, that makes arrangements for establishing a fund that promotes protection of biodiversity and sustainable development of forest resources; and the *Environment Management Act No. 20, of 2004* that provides for legal and institutional framework for sustainable management of the environment, prevention and control of pollution (Pastory 2022).
- In the Republic of Zambia, the legal basis for DRR is the *Disaster Management Act No. 13, of 2010* that establishes and provides for the maintenance and operation of a system for anticipation, preparedness, prevention, coordination, mitigation and management of disasters (UNDRR n.d.). This Act was put into action by other document designated as National Disaster Management Policy of 2015, that promotes the sustainable development among vulnerable communities and improves their resilience. Ecosystem and biodiversity conservation are regulated in separate documents such as the *Environment Management Act No. 12, of 2011* covering environment planning, biosecurity, ecosystem preservation, protected areas, and soil conservation and improvement; *The Forest Act No. 4, of 2015* covering climate change, forest management, protection and conservation, and afforestation and/or reforestation.
- In the Republic of Zimbabwe, the DRR system is governed by the *Civil Protection Act of 1989*, focussed on civil protection and the regulation and funding of civil protection in times of disaster (IDRL 2021). Further statutory tools incorporating ecosystem and biodiversity, are the *Environment Management Act No. 12, of 2002* covering conservation and improvement of environment, general principles of environmental and functions of minister and environment management board; The National Contingency Plan 2012–2013 and the National Climate Policy 2016.

## Discussion

In a few of the SADC member states, Eco-DRR-related policies have existed before SADC region was established in 1992, namely the Kingdom of Eswatini with *Natural Resources Act No. 72 of 1951*, the Republic of Namibia with the Nature Conservation Ordinance *Law No 4 of 1975* and the Republic of Zimbabwe with Civil Protection of 1989. After the SADC was formed, most of the member states started to develop their own instruments relevant to Eco-DRR, comprising acts, laws, policies, strategies and plans. It is customary for countries to possess a DRM system that is supported by one or more laws, policies, legislations and frameworks. The importance of the national Eco-DRR policy documents is to recognise the intrinsic links between DRR, CCA and the role of ecosystems in addressing these risks.

Over time, SADC member states have been creating new policies and improving the existing ones according to the country’s type of hazard and the international standards of Eco-DRR. When the Hyogo Framework for Action for DRR 2005–2015 was adopted, it set out priorities to help countries to achieve disaster resilience by encouraging the establishment of national platforms and strengthening disaster governance (Calkins 2015). Some amendments in Acts were done, as well as new policies, plan and strategies were developed. Towards this end, there has been significant additional effort at global, national, sub-national and community levels, to fortify legal, institutional and practical measures to minimise the overwhelming effects of disasters resulting in a convergence on agreements on DRR, development finance, sustainable development and climate change across their inter-related policy areas. However, for the analysed Eco-DRR documents, there is a lack of standardised and technical guidelines for the design and implementation of Eco-DRR policies, plans and strategies for the SADC region. Additionally, the inclusion of Eco-DRR specific aspects in biodiversity conservation became more apparent in recent Eco-DRR policy documents that resulted from increasing DRM practice. However, this inclusion comes in fragmented legislative documents depending on tutelary institution.

Some states such as the Republic of Mauritius, Comoros and Kingdom of Eswatini have developed specific activities towards Eco-DRR. However, they have not turned them into laws or policies. The activities range from programmes, plans of actions, meetings, conferences and capacity development. Despite the positive trend in implementing Eco-DRR policies in the SADC countries, some governments have not revised or updated their policies. Zimbabwe has the most recent policy framed in 2016, while Namibia has a policy framed in 2009 and Lesotho still follows the one it adopted in 1998.

The analysis of the policies revealed that SADC member states are focussed in their internal Eco-DRR issues, leaving aside transboundary issues. All the analysed Eco-DRR policy documents were aiming at regulating internal issues only. This is true even for countries that share resources and have common disaster profiles; for example, DRC, Zambia, Zimbabwe, Malawi, Botswana, Namibia, Angola, South Africa, Lesotho, Eswatini and Mozambique. This issue was also pointed out by Tau et al. (2016) who stated that the lack of collaboration among southern African countries on DRM dates back in the history regardless of common cross border and similar disaster characteristics. The authors justified the challenge in collaboration as a result of different stages in development of policies, frameworks and practical measures of the countries. The Eco-DRR collaboration in southern African countries is instead entrusted to SADC as regional body, private companies and civil society.

The southern Africa DRR Plan 2012–2014 recognises the need to develop a long-term strategic approach that helps articulate funding and programme priorities to allow for comprehensive disaster programming that reduces future humanitarian needs. From the analyses, it was found that some of the southern African member states develop short-term plans.

While many countries have legislation for DRR and environmental issues, there seem to be relatively few countries that have legislation that promotes eco-DRR explicitly, suggesting a gap between regional policy aspirations and country-level legislation and policies.

Overall, it is evident that there is a regional interest and demand to apply and standardise ecosystem-based approaches and natural or green infrastructure solutions as conservation, restauration, adaptation and management strategies and policies to reduce disaster risks.

## Conclusion

This article assessed statutory and policy-based eco-disaster risk reduction mechanisms in SADC member states. The inefficiencies in governance and Eco-DRR policy making in SADC member states are evident because of the lack of empowerment of the existing institutions and creation of networks that are driven by southern African institutions. Another fact to consider is the creation of short-term plans and/or strategies that doesn’t help to articulate funding and programme priorities. Moreover, there is a lack of standardised, technical guidelines for designing and utilisation of ecosystem-based measures for reducing disaster risks. This constrains the engineering community from further replicating and implementing such measures. Furthermore, the updates of the existing Eco-DRR policies and legislations are not regular. They are performed following the occurrence of a catastrophic event. This demonstrates how the eco-disaster risk reduction is not a top priority agenda for some member states, especially those with high rate of poverty.

For the analysis presented in the current framework, it can also be concluded that there is a lack of indicators and follow-up strategies on the implementation of the existing policies, plans or strategies despite the existence of guidelines and recommendation frameworks. The identified variables underpinning the policies are generally common, ranging from conservation, restoration, adaptation and management.

Furthermore, the lack of transboundary collaboration in Eco-DRR issues is pervasive in the SADC member states (rather end with a stronger focus on the possible future of Eco-DRR policies and instruments).
